# Gonadotropin Suppression During Mini‐Puberty as an Early Biomarker of Classic 21‐Hydroxylase Deficiency

**DOI:** 10.1002/edm2.70317

**Published:** 2026-09-02

**Authors:** Ryosei Iemura, Yuri Suzuki, Maki Gau, Ryuta Orimoto, Haruki Yamano, Hisae Nakatani, Shizuka Kirino, Yoko Saito, Eriko Adachi, Atsumi Tsuji‐Hosokawa, Kenichi Kashimada, Kei Takasawa

**Affiliations:** ^1^ Department of Pediatrics and Developmental Biology Institute of Science Tokyo Tokyo Japan; ^2^ Department of Pediatrics Chiba Kaihin Municipal Hospital Chiba Japan; ^3^ Department of Life Science and Bioethics Institute of Science Tokyo Tokyo Japan; ^4^ Division of Endocrinology and Metabolism National Center for Child Health and Development Tokyo Japan

**Keywords:** 21‐hydroxylase deficiency, gonadotropin, newborn screening

## Abstract

**Introduction:**

Newborn screening (NBS) for congenital adrenal hyperplasia caused by 21‐hydroxylase deficiency (21OHD) relies on elevated 17‐hydroxyprogesterone (17OHP) levels but is limited by a high false‐positive rate and difficulty in distinguishing classic from non‐classic forms. To evaluate whether the suppression of serum luteinizing hormone (LH) and follicle‐stimulating hormone (FSH) during mini‐puberty can serve as an early biomarker of classic 21OHD.

**Methods:**

We conducted a retrospective cohort study of 37 infants evaluated for suspected 21OHD between 2013 and 2025. Subjects were classified as classic (C), non‐classic (NC), or false‐positive (FP) based on biochemical and genetic confirmation. Neonatal serum LH and FSH levels were compared among groups, and receiver operating characteristic (ROC) analyses were performed.

**Results:**

LH and FSH levels were significantly suppressed in classic 21OHD compared with NC and FP cases (*p* < 0.001). Gonadotropin concentrations demonstrated a stepwise pattern (C<NC<FP), although overlap limited the discrimination of non‐classic disease. ROC analyses identified optimal cutoff values of 0.3 mIU/mL for LH (sensitivity 89.5%, specificity 83.3%) and 1.3 mIU/mL for FSH (sensitivity 94.7%, specificity 100%) for identifying classic 21OHD. Suppression was observed in both sexes and was particularly informative in male neonates.

**Conclusions:**

Suppressed LH and FSH levels during the neonatal period reflect the attenuation of mini‐puberty and represent a characteristic endocrine feature of classic 21OHD. Gonadotropin measurement may provide a clinically accessible adjunct to NBS for the early identification of severe disease, particularly in male neonates and in the evaluation of 46,XX disorders/differences in sex development.

## Graphical abstract

To prevent life‐threatening adrenal crisis and to help perform appropriate sex assignment in affected female patients, newborn screening for 21‐hydroxylase deficiency (21OHD) relies on 17‐hydroxyprogesterone but has a high false‐positive rate. Neonatal LH and FSH suppression during mini‐puberty clearly distinguished classic 21OHD from non‐classic and false‐positive cases with excellent diagnostic accuracy. Because male neonates often lack overt signs and 46,XX cases need careful work‐up, gonadotropin measurement offers a simple, accessible tool to flag classic disease early and guide prompt treatment.

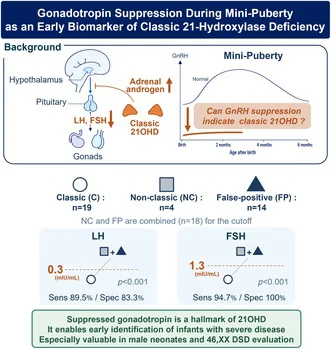

## 1 Introduction

Congenital adrenal hyperplasia (CAH) is an inherited disorder caused by the loss or severely impaired activity of steroidogenic enzymes involved in cortisol biosynthesis. 21‐hydroxylase deficiency (21OHD) accounts for over 90% of CAH cases and represents a spectrum of autosomal recessive disorders caused by variants in the *CYP21A2* gene [1, 2]. The enzyme 21‐hydroxylase catalyses key steps in the biosynthesis of cortisol and aldosterone, and its deficiency leads to accumulation of steroid precursors and excess adrenal androgen production. 21OHD presents with a spectrum of phenotypes. A severe form with a concurrent defect in aldosterone biosynthesis (salt‐wasting; SW) and a form with apparently normal aldosterone biosynthesis (simple virilizing; SV) are together termed classic 21OHD. There is also a mild, nonclassic form that may be asymptomatic or associated with signs of postnatal androgen excess. Approximately 75% of patients with classic 21OHD exhibit salt wasting due to aldosterone deficiency, leading to potentially life‐threatening dehydration and electrolyte imbalance during the neonatal period [1].

Affected female foetuses are exposed to excess adrenal androgens from approximately the seventh week of gestation, resulting in virilized external genitalia at birth [2]. In contrast, male infants typically appear normal at birth, making early diagnosis more difficult in the absence of newborn screening. These represent major clinical problems in both the SW and SV forms. To prevent life‐threatening adrenal crisis and to help perform appropriate sex assignment in affected female patients, newborn screening (NBS) based on 17‐OHP quantification programs for the classical form of CAH have been introduced in numerous countries. In Japan, the NBS for CAH was introduced in 1989 and has detected 21OHD in approximately 1 in 18,000–19,000 live births [3]. Although the effectiveness of newborn mass screening for the diagnosis of 21OHD is well established, the high frequency of false positives (i.e., a low positive predictive value) remains the most significant problem [4]. In addition, the affected individuals include asymptomatic patients with the NC form, which does not require treatment, and it is sometimes difficult to distinguish them from patients with the SW and SV form, which is at higher risk of salt‐wasting.

To address these issues, we focused on gonadotropin levels during the neonatal period, specifically during the transient postnatal surge known as mini‐puberty, in which gonadotropin levels temporarily increase in early life. After birth, the hypothalamic–pituitary–gonadal axis is activated when oestrogen is eliminated with placental separation and a hormone profile which reaches pubertal levels is established. These changes are called mini‐puberty [5]. However, in infants with 21OHD, this axis may be suppressed due to elevated adrenal androgens present since the fetal period, potentially leading to decreased levels of LH and FSH. In the present study, based on this pathophysiology, we investigated whether suppressed gonadotropin levels could serve as an early biomarker for the diagnosis of 21OHD and for identifying the severity of salt‐wasting.

## 2 Material and Methods

### 2.1 Patients

We conducted a retrospective review using the medical records of patients who underwent detailed evaluation for 21OHD at Institute of Science Tokyo based on newborn screening (NBS) results or atypical external genitalia between 2013 and 2025, with measurements of serum concentrations of gonadal hormones (LH and FSH). True 21OHD cases were differentiated from false positive cases using biochemical findings such as urinary steroid profiles and rapid ACTH stimulation tests, and all true cases were confirmed endocrinologically and genetically. Genetic testing was performed using long‐read sequencing (LRS) technology [6]. Cases in which the initial examination was performed after 4 months of age, when the gonadotropin peak during mini‐puberty is considered to have passed, were excluded [7]. In this study, serum LH and FSH values were measured by electrochemiluminescence immunoassay (ECLIA), and values below the detection limit were replaced with the lower limit of detection.

### 2.2 Statistics

Statistical analyses were performed using R software (EZR version 1.70; Saitama Medical Center, Jichi Medical University, Saitama, Japan). Continuous variables are presented as medians with ranges. Serum concentrations of LH and FSH were compared among infants with classic 21OHD, non‐classic 21OHD and false‐positive cases using the Kruskal–Wallis test. When significant differences were observed, pairwise comparisons were performed using the Mann–Whitney *U* test with Bonferroni correction. Comparisons between two groups were also performed using the Mann–Whitney *U* test. ROC curve analyses were conducted to determine the optimal cutoff values for LH and FSH in identifying classic 21OHD, and diagnostic performance was evaluated by calculating sensitivity and specificity. A two‐sided *p* value < 0.05 was considered statistically significant.

### 2.3 Ethics

This study was approved by the ethics committee of the Institute of Science Tokyo (M2023‐336). Clinical data were linked to the patients' disease classifications, and previously obtained genetic test results were used when available. Informed consent was obtained through an opt‐out procedure in accordance with institutional guidelines. Genetic counselling was available upon request to families who wished to discuss prior results.

## 3 Results

A total of 37 infants (16 males) evaluated for suspected 21‐hydroxylase deficiency (21OHD) were included in the analysis. Individual‐level clinical, biochemical and genetic characteristics are summarized in Table 1, including phenotype classification, sex, age at blood sampling, serum LH and FSH concentrations during the neonatal period, gestational age, birth weight, newborn screening results, ACTH stimulation test findings and confirmed *CYP21A2* genotypes. Based on comprehensive endocrine and genetic evaluations, subjects were classified as having classic 21OHD (C, *n* = 19), non‐classic 21OHD (NC, *n* = 4), or false‐positive newborn screening results (FP, *n* = 14). All diagnoses in the C and NC groups were genetically confirmed using LRS. As shown in Table 1, significant differences were observed among the three groups in gestational age, birth weight and age at hormonal sampling (Kruskal–Wallis test; gestational age: *p* = 4.2 × 10^−4^; birth weight: *p* = 9.9 × 10^−4^; age at sampling: *p* = 2.1 × 10^−6^). Blood sampling was performed earlier in the C group (median 9.0 days) than in the NC (16.5 days) and FP groups (39.5 days). Within the C group, age at sampling did not differ significantly between males and females (Mann–Whitney *U* test, *p* = 0.098).

**TABLE 1:** Clinical characteristics of patients.

Phenotype	Sex	Age	LH (mIU/mL)	FSH (mIU/mL)	Genotype		Gestational age (week)	Birth weight (g)	Newborn screening			Na	K	ACTH stimulation test	
									17OHP (ng/mL)_LC‐MS/MS	21DOF (ng/mL)_LC‐MS/MS	17OHP (ng/mL)_ELISA			Basal 17OHP (ng/mL)	Peak 17OHP (ng/mL)
C	F	6	0.1	0.3	Large Deletion	I173N	39.4	3428	—	—	> 100	132	5.3	—	—
C	F	7	0.2	1	I2G	R484fs、H63L	40.0	3730	—	—	78.6	130	6.8	—	—
C	F	8	0.1	0.1	I173N	R484fs	39.4	3126	—	—	10.4	136	5.1	—	—
C	F	9	0.1	0.2	E6 cluster	R357W	37.3	2602	89.43	3.66	—	127	7.6	—	—
C	F	10	0.4	0.5	CH1	I173N	40.4	2810	7.59	1.73	—	135	5.6	—	—
C	F	6	0.3	0.3	Q319^a^	R357W	40.3	3324	80.26	24.93	—	139	5.3	—	—
C	F	5	0.3	0.3	I173N	Q319^a^^R357W	37.4	1942	32.29	23.32	—	141	4.6	—	—
C	F	6	0.3	2.4	CH2	Splicing variant	36.0	2784	46.96	23.9	—	140	5.2	—	—
C	F	9	0.3	0.3	I2G	I173N	39.4	3618	8.95	11.74	—	138	5.7	—	—
C	M	10	0.2	1	I173N	I173N	39.3	3046	—	—	361	125	6.3	—	—
C	M	6	0.2	1	CH1	V282L^R357W	40.3	2740	—	—	169.8	137	5.7	—	—
C	M	12	0.2	1	I2G	Splicing variant	38.0	3032	—	—	> 100	139	5.8	—	—
C	M	9	0.2	0.1	CH2a	R357W	38.0	2650	—	—	160.9	132	6.8	—	—
C	M	7	0.1	0.1	CH1	I173N^R357W	41.0	3434	—	—	> 300	136	5.3	—	—
C	M	26	2.2	1.3	I173N	I173N	39.3	3200	3.06	1.27	—	135	5.3	—	—
C	M	10	0.3	0.3	I2G	I2G	40.9	3422	120.35	22.35	—	128	7.3	—	—
C	M	5	0.3	0.3	I173N	Q319^a^^R357W	37.4	2694	24.67	6.35	14.1	141	4.4	—	—
C	M	9	0.3	0.3	I173N	R484fs	38.6	3524	20.22	0.72	—	128	6.6	—	—
C	M	12	0.3	0.3	I2G	I2G	38.3	3244	46.9	21.21	—	131	6.5	—	—
*Median*		9	0.3	0.3			39.3	3126				135	5.7		
NC	F	16	0.2	5.4	CH1	P31L^a^	40.4	3058	—	—	10.9	136	5.8	19.8	151.2
NC	F	118	0.1	4.8	I2G	V282L^a^	39.3	3588	—	—	7.8	142	4.9	10.9	55.2
NC	M	17	6.2	1.8	c.‐[126C>T; −113G>A]^a^	I2G	40.9	3194	8.28	10.01	—	139	5.3	11.8	25
NC	M	12	11.2	11.2	c.‐[126C>T; −113G>A]^a^	I2G	38.0	3030	4.3	1.37	—	141	5.3	30	40
*Median*		16.5	3.2	5.1			39.9	3126				140	5.3	15.8	47.6
FP	F	32	4.5	88.5	—	—	37.1	2760	—	—	5.3	139	5.2	4.2	6.1
FP	F	46	1.9	4.6	—	—	37.0	2360	—	—	8.6	139	5.2	4.2	8.7
FP	F	34	1.1	27.9	—	—	37.7	2460	—	—	6.1	140	5.8	7.0	8.8
FP	F	63	0.6	31.9	—	—	36.1	2586	—	—	4.8	140	4.8	2.5	3.2
FP	F	17	0.4	3.5	—	—	37.1	3024	—	—	10.3	141	4.8	2.5	4.2
FP	F	38	0.5	7.1	—	—	37.4	3000	—	—	3.6	137	5.6	0.8	1.9
FP	F	41	0.2	10.4	—	—	38.6	2624	—	—	3.4	141	5.1	1.2	1.5
FP	F	58	0.6	12.3	—	—	37.0	2500	—	—	6.4	136	5.2	< 0.1	2.1
FP	F	37	3.6	49.3	—	—	37.6	2740	—	—	4.5	140	4.6	3.6	6.7
FP	F	52	8	43	—	—	37.3	2014	2.7	0	—	139	5.2	1.5	ND
FP	M	48	5.2	3.1	—	—	37.1	2565	—	—	5.8	137	5.7	1	2.7
FP	M	65	2.1	1.8	—	—	36.1	2606	—	—	7.1	138	4.6	1	2
FP	M	38	2.6	3.4	—	—	37.0	2870	—	—	3	142	4.9	1.6	2
FP	M	26	2.1	1.6	—	—	38.6	2694	3.4	0.25	—	140	5.3	4.3	5.2
*Median*		39.5	2	8.8			37.1	2615				140	5.2	2.5	3.2
*p* value*		2.13.E‐06	6.56.E‐04	1.76.E‐06			4.17.E‐04	9.87.E‐04				4.41E‐03	2.47E‐02		
C+NC vs. FP		5.79.E‐06	3.97.E‐04	1.59.E‐05			1.14.E‐04	2.63.E‐04				1.34E‐02	9.82E‐03		
C vs. NC+FP		5.07.E‐07	2.31.E‐04	3.06.E‐07			8.08.E‐03	1.12.E‐02				1.08E‐03	1.07E‐02		

Abbreviations: 17OHP, 17‐hydroxyprogesterone; 21DOF, 21‐deoxycortisol; C, classic; FP, false‐positive newborn screening; NC, non‐classic.

^a^ *CYP21A2* variants predictive of non‐classical congenital adrenal hyperplasia.

* *p* values were calculated using the Kruskal–Wallis test and Mann–Whitney *U* test.

**FIGURE 1:**
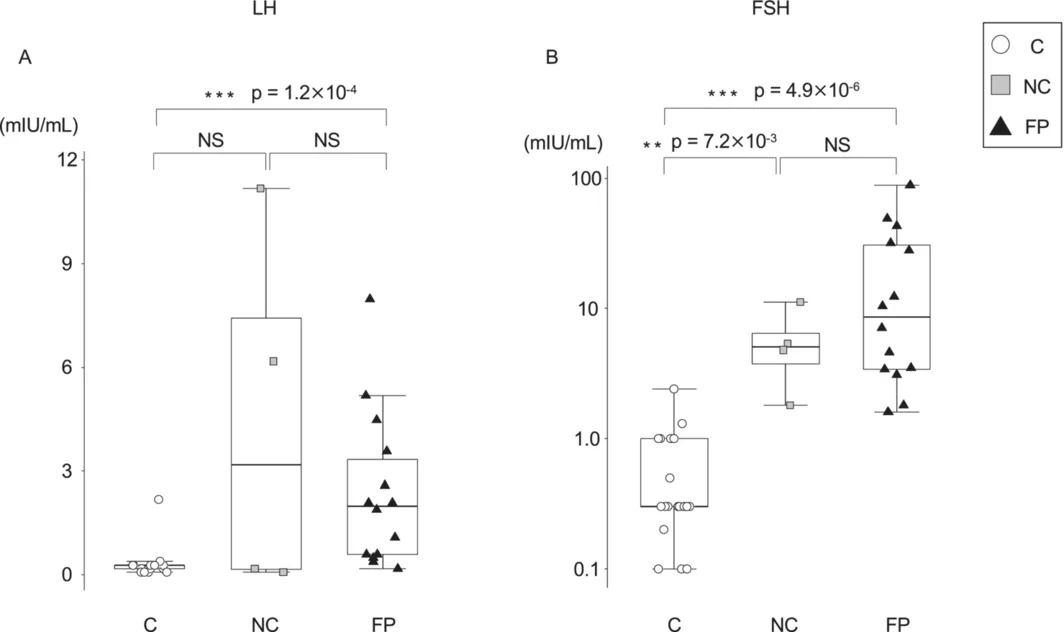
Suppression of neonatal gonadotropins in classic 21‐hydroxylase deficiency. Serum luteinizing hormone (LH) (A) and follicle‐stimulating hormone (FSH) (B) concentrations measured during the neonatal period in infants evaluated for suspected 21‐hydroxylase deficiency (21OHD). Subjects were classified as classic 21OHD (C), non‐classic 21OHD (NC), or false‐positive newborn screening results (FP). Infants with classic 21OHD exhibited markedly suppressed LH and FSH concentrations compared with NC and FP cases. Gonadotropin levels showed a stepwise distribution across phenotypes (C<NC<FP), although partial overlap between NC and FP cases was observed. Group comparisons were performed using the Kruskal–Wallis test, followed by pairwise Mann–Whitney *U* tests with Bonferroni correction. Horizontal bars indicate median values. Asterisks indicate significant differences (*p < 0.05, **p < 0.01, ***p < 0.001); NS indicates not significant.

Inspection of individual data in Table 1 revealed marked suppression of serum LH and FSH concentrations in infants with classic 21OHD compared to NC and FP cases. Nearly all classic cases showed LH values close to or below the lower detection limit and uniformly low FSH concentrations, whereas NC and FP cases exhibited a broader distribution of gonadotropin values. Group‐wise comparisons confirmed these observations (Figure 1). Serum FSH levels were significantly lower in the C group than in the NC group (*p* = 7.2 × 10^−3^) and the FP group (*p* = 4.9 × 10^−6^), while no difference was observed between the NC and FP groups (*p* = 1.0). For LH, a significant difference was detected between the C and FP groups (*p* = 1.2 × 10^−4^), whereas comparisons involving the NC group did not reach statistical significance. Taken together with the individual profiles shown in Table 1, these results indicate a consistent stepwise pattern of gonadotropin suppression across phenotypes (C<NC<FP), although overlap between NC and FP cases limited the discrimination of non‐classic disease.

**FIGURE 2:**
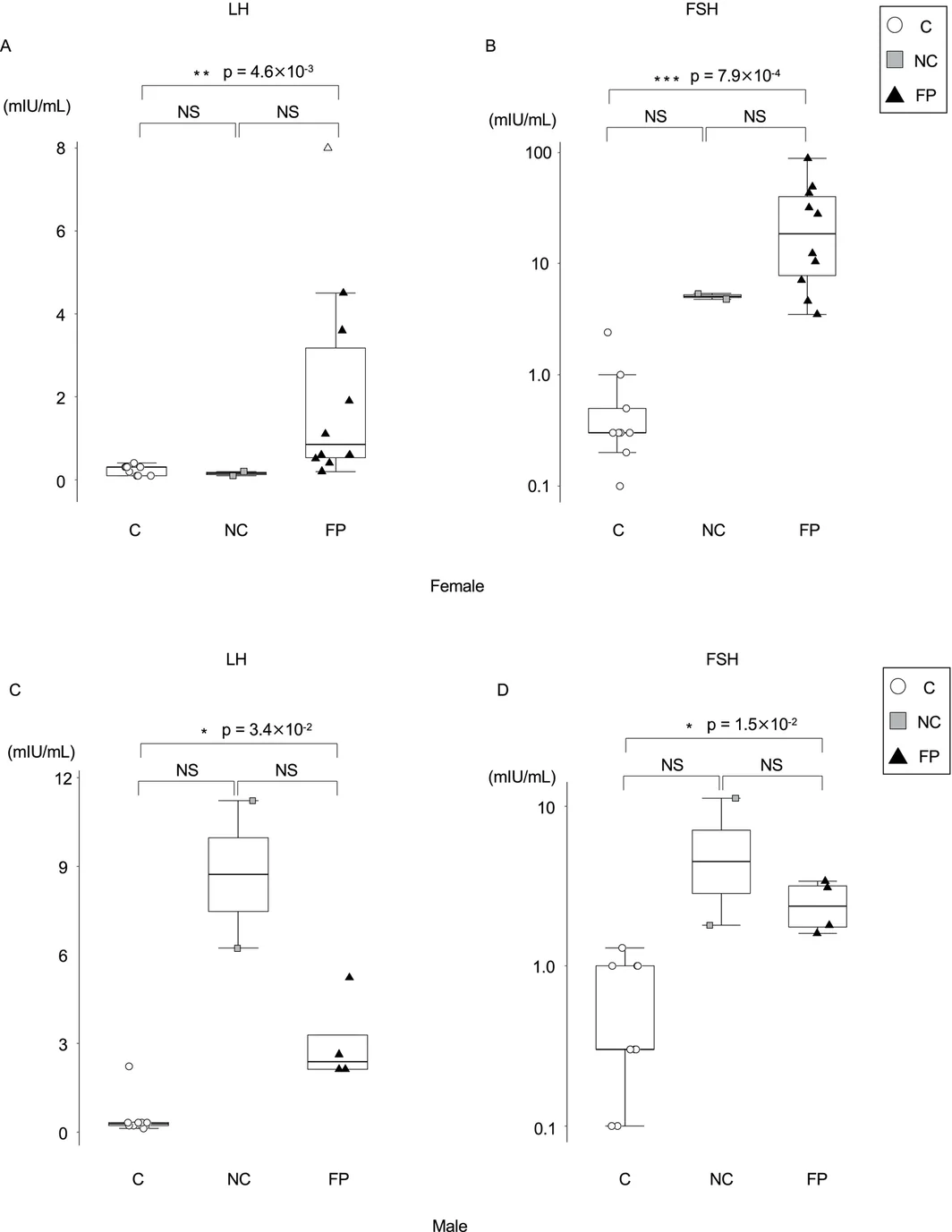
Sex‐stratified distribution of LH and FSH during mini‐puberty. Serum gonadotropin concentrations stratified by sex in infants evaluated for suspected 21OHD. (A) LH and (B) FSH levels in female infants. (C) LH and (D) FSH levels in male infants. In both sexes, infants with classic 21OHD demonstrated significantly lower gonadotropin concentrations than those with false‐positive newborn screening results. Non‐classic cases showed intermediate values, particularly among male infants. Statistical analysis was performed using the Kruskal–Wallis test with Bonferroni‐corrected pairwise comparisons. Horizontal bars represent median values. Asterisks indicate statistically significant differences; NS indicates not significant.

The sex‐stratified evaluation of individual cases in Table 1 demonstrated that suppression of LH and FSH in classic 21OHD was observed in both female and male infants. In both sexes, gonadotropin concentrations were significantly lower in the C group than in the FP group (*p* < 0.05 for all comparisons, Figure 2). Notably, among male infants, individual NC cases showed gonadotropin values that were intermediate between those of classic and FP cases. While the overlap remained, LH and FSH levels in male NC cases tended to be higher than those observed in most classic cases, suggesting a partial phenotypic separation when interpreted at the individual level.

**FIGURE 3:**
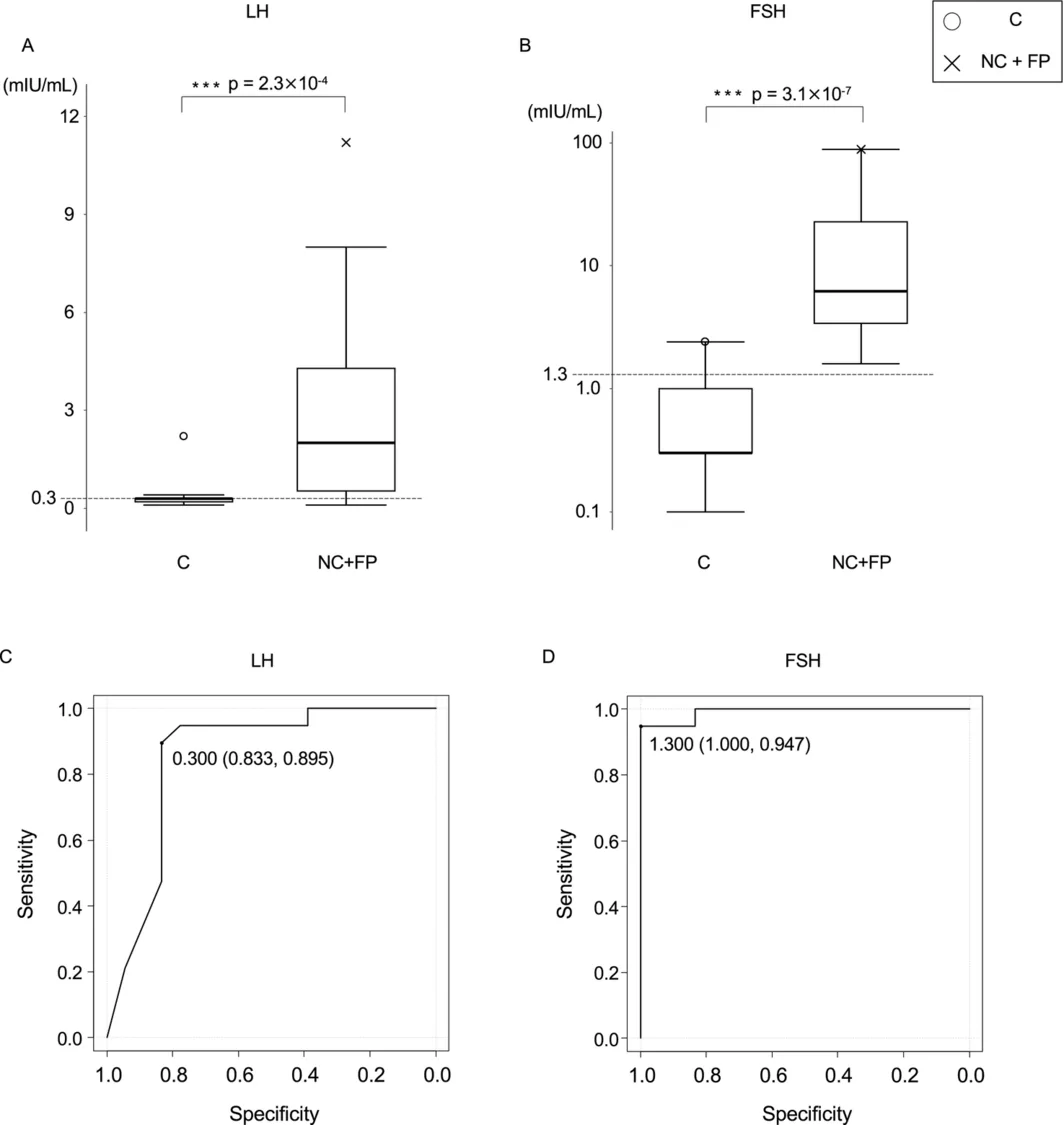
Diagnostic performance of LH and FSH for identifying classic 21OHD. Comparison of gonadotropin levels between infants with classic 21OHD (C) and those without classic disease (NC+FP). (A) Serum LH levels and (B) serum FSH levels were significantly lower in the C group. (C) Receiver operating characteristic (ROC) curve for LH. (D) ROC curve for FSH. ROC analysis identified optimal cutoff values of 0.3 mIU/mL for LH and 1.3 mIU/mL for FSH. The LH cutoff yielded a sensitivity of 89.5% and specificity of 83.3%, whereas the FSH cutoff achieved a sensitivity of 94.7% and specificity of 100%. Group comparisons were performed using the Mann–Whitney *U* test.

**FIGURE 4:**
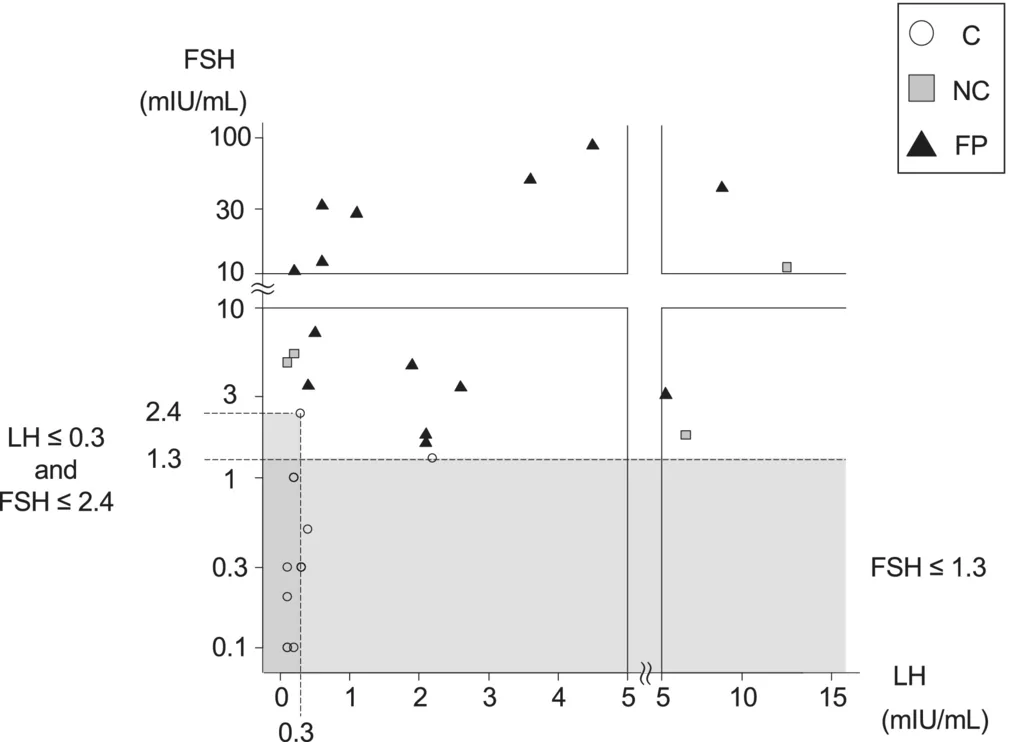
Combined LH–FSH classification rule for detecting classic 21OHD. Scatter plot of serum LH and FSH concentrations in infants classified as classic 21OHD (C), non‐classic 21OHD (NC), or false‐positive screening results (FP). Based on ROC‐derived thresholds, an axis‐parallel decision rule was constructed to identify classic disease. Infants were classified as having classic 21OHD when FSH ≤ 1.3 mIU/mL, or when LH ≤ 0.3 mIU/mL and FSH ≤ 2.4 mIU/mL. This rule achieved 100% sensitivity (19/19) and 100% specificity (18/18) in this cohort.

To evaluate clinical utility, the C group was compared with the combined NC+FP group. Both LH and FSH levels were significantly lower in classic cases (Figure 3A,B; *p* = 2.3 × 10^−4^ and *p* = 3.1 × 10^−7^, respectively). ROC analysis identified optimal cutoff values of 0.3 mIU/mL for LH and 1.3 mIU/mL for FSH, providing high sensitivity and specificity for identifying classic 21OHD; LH achieved a sensitivity of 89.5% and a specificity of 83.3%, while FSH demonstrated a sensitivity of 94.7% and a specificity of 100% (Figure 3C,D). These cutoff values were concordant with the individual distributions observed in Table 1, in which the majority of classic cases clustered below these thresholds. Furthermore, to discriminate the C group from non‐C cases (FP+NC), we constructed an axis‐parallel rule using serum FSH and LH. The model classified cases as C if FSH ≤ 1.3, or if LH ≤ 0.3 and FSH ≤ 2.4; otherwise, cases were classified as non‐C, achieving a sensitivity of 100% and a specificity of 100% (Figure 4).

## 4 Discussion

In this study, we demonstrated that serum LH and FSH concentrations during the neonatal period are markedly suppressed in infants with classic 21OHD. This pattern was consistently observed at the individual level and enabled the discrimination of classic cases from non‐classic and false‐positive cases detected through NBS. These findings indicate that the suppression of gonadotropin secretion during mini‐puberty represents a characteristic endocrine feature of classic 21OHD.

Mini‐puberty refers to the transient postnatal activation of the hypothalamic–pituitary–gonadal (HPG) axis that occurs mainly within the first few days or weeks until 6 months of life, following the withdrawal of placental sex steroids after birth. During this period, gonadotropin secretion increases, resulting in elevated LH and FSH levels and subsequent gonadal steroid production in both sexes [8, 9, 10]. Although the biological role of mini‐puberty has not been fully elucidated, current evidence supports its value as a physiological window for assessing spontaneous HPG axis function, rather than as a determinant of fixed developmental outcomes.

In male infants, mini‐puberty is associated with increased testosterone production, testicular and penile growth and activation of Sertoli and Leydig cell functions [11, 12]. In females, activation of the postnatal HPG axis leads to transient ovarian activity, follicular maturation and fluctuating estradiol levels, although the downstream physiological consequences are less clearly defined. Importantly, several comprehensive reviews emphasize that mini‐puberty provides a unique opportunity for early endocrine evaluation, particularly in infants with suspected disorders of sex development (DSD) or hypogonadism, without the need for stimulation testing.

The marked suppression of LH and FSH observed in infants with classic 21OHD suggests attenuation of postnatal HPG axis activation. In classic 21OHD, excessive adrenal androgen exposure begins in fetal life and persists into the neonatal period owing to impaired cortisol synthesis and chronic ACTH stimulation. Sustained androgen excess is therefore likely to exert negative feedback at the hypothalamic and pituitary levels, limiting the postnatal rise in gonadotropin secretion that normally characterizes mini‐puberty. This interpretation is supported by the observation that gonadotropin suppression is most pronounced in classic cases and less evident in non‐classic cases, which are characterized by milder enzymatic impairment and lower cumulative androgen exposure. Accordingly, the suppression of mini‐puberty in 21OHD appears to reflect disease severity rather than serve as a binary diagnostic marker.

Although we were unable to reliably discriminate between classic and non‐classic 21OHD based solely on gonadotropin levels, this study has important clinical implications. Among infants identified through NBS based on elevated 17OHP levels, where false‐positive results are common, suppressed LH and FSH concentrations may serve as early indicators of classic 21OHD requiring prompt therapeutic intervention. A major advantage of gonadotropin assessment is its clinical accessibility. Unlike 17OHP, which is a specialized assay with a high false‐positive rate that often requires time‐consuming confirmatory testing [4, 13, 14], LH and FSH are widely available routine endocrine tests. Measurement of these hormones during the neonatal period may therefore facilitate earlier identification of infants at high risk of salt‐wasting crisis [1, 15], allowing the timely initiation of glucocorticoid and mineralocorticoid replacement before the onset of potentially life‐threatening dehydration and electrolyte imbalance. This clinical value is particularly relevant because early symptoms of adrenal insufficiency in neonates are often nonspecific, such as poor vitality or inadequate weight gain. Moreover, hyperpigmentation due to excess ACTH lacks quantitative diagnostic criteria and may be overlooked by those without clinical experience. Furthermore, in healthcare settings where NBS programs for CAH are not fully implemented or optimized, gonadotropin measurements may provide a practical adjunct to support early diagnosis and intervention.

Early diagnosis of classic 21OHD is especially challenging in male neonates who typically exhibit no overt genital abnormalities at birth [16]. In this context, suppressed LH and FSH levels during the neonatal period may provide additional biochemical signals, suggesting severe disease and warranting close monitoring and early treatment initiation. From a DSD perspective, these findings are also relevant to female infants with atypical external genitalia. Because CAH accounts for the majority of 46,XX DSD cases [17], the presence of gonadotropin suppression supports prenatal androgen excess, which is consistent with CAH. Conversely, the absence of suppression may help exclude CAH and prompt consideration of alternative diagnoses, including disorders of gonadal differentiation (e.g., testicular DSD with SRY positivity or SOX9 duplication, ovotesticular DSD and primary ovarian insufficiency due to mutations in the FSH receptor, NR5A1 or WT1), syndromic conditions, and structural anomalies of the genital tract [18]. Thus, assessment of LH and FSH levels during mini‐puberty may contribute to refining the differential diagnosis in neonates presenting with DSD.

Consistent with previous observations, gonadotropin measurements alone did not allow for a clear discrimination between non‐classic 21OHD and false‐positive cases. However, a key strength of the present study lies in the use of LRS–based genetic analysis [6], which enabled the accurate classification of non‐classic cases through established genotype–phenotype correlations [19]. This approach ensured robust diagnostic assignment and supports the interpretation that gonadotropin suppression should be viewed primarily as an indicator of disease severity rather than as a definitive diagnostic marker for non‐classic 21OHD.

Although mini‐puberty is associated with early gonadal maturation and genital growth, its long‐term clinical significance remains unclear. Current evidence linking alterations in mini‐puberty to later fertility [12, 20] or neurodevelopment [21, 22, 23] is limited and largely observational. Therefore, any potential contribution of suppressed mini‐puberty to long‐term outcomes in patients with 21OHD should be interpreted with caution. This study has several limitations, including its retrospective, single‐centre design and the relatively small overall sample size, particularly the small number of non‐classic cases, which may limit the generalizability of our findings. Differences in age at sampling reflect real‐world clinical practice following NBS and may influence gonadotropin concentrations. Moreover, because the proposed LH and FSH cutoff values were derived and evaluated within the same cohort, they may be susceptible to overfitting, and their diagnostic performance requires confirmation in independent populations. Validation of these cutoff values in larger, prospective, multicentre cohorts is therefore needed before gonadotropin measurement can be recommended as a routine adjunct to newborn screening programs. Nevertheless, the consistent suppression observed across classic cases supports the robustness of the findings.

## 5 Conclusion

In conclusion, suppressed gonadotropin secretion during the neonatal period reflects the attenuation of mini‐puberty and represents a characteristic endocrine feature of classic 21‐hydroxylase deficiency. While gonadotropin measurements do not permit definitive discrimination of all CAH phenotypes, they provide clinically meaningful adjunctive information for the early identification of infants with severe disease, particularly in male neonates and in the evaluation of 46, XX DSD. While the long‐term effects of suppressed mini‐puberty warrant further investigation, assessment of LH and FSH levels during early infancy may complement established biochemical and genetic approaches, supporting timely diagnosis and intervention in classic 21OHD.

## Author Contributions

**Atsumi Tsuji‐Hosokawa:** data curation, investigation. **Hisae Nakatani:** data curation, investigation. **Kei Takasawa:** conceptualization, funding acquisition, project administration, supervision, writing – review and editing. **Shizuka Kirino:** data curation, investigation, visualization. **Ryuta Orimoto:** data curation, investigation. **Eriko Adachi:** resources, supervision. **Ryosei Iemura:** conceptualization, data curation, formal analysis, investigation, methodology, visualization, writing – original draft, writing – review and editing. **Maki Gau:** data curation, investigation. **Yoko Saito:** data curation, investigation. **Haruki Yamano:** data curation, investigation. **Kenichi Kashimada:** resources, supervision. **Yuri Suzuki:** data curation, investigation.

## Funding

This work was supported by a grant from the Japan Agency for Medical Research and Development (AMED) (No. 23ek0109630h0001).

## Conflicts of Interest

The authors declare no conflicts of interest.

## Data Availability

The data that support the findings of this study are available on request from the corresponding author. The data are not publicly available due to privacy or ethical restrictions.
